# Interfield dysbalances in research input and output benchmarking: Visualisation by density equalizing procedures

**DOI:** 10.1186/1476-072X-7-48

**Published:** 2008-08-25

**Authors:** Beatrix Groneberg-Kloft, Carolin Kreiter, Tobias Welte, Axel Fischer, David Quarcoo, Cristian Scutaru

**Affiliations:** 1 Otto-Heubner-Centre, Charité-Universitätsmedizin Berlin, Free University Berlin and Humboldt-University Berlin, Germany; 2 Department of Respiratory Medicine, Hannover Medical School, Hannover, Germany; 3 Institute of Occupational Medicine, Charité-Universitätsmedizin Berlin, Free University Berlin and Humboldt-University Berlin, Germany

## Abstract

**Background:**

Historical, social and economic reasons can lead to major differences in the allocation of health system resources and research funding. These differences might endanger the progress in diagnostic and therapeutic approaches of socio-economic important diseases. The present study aimed to assess different benchmarking approaches that might be used to analyse these disproportions. Research in two categories was analysed for various output parameters and compared to input parameters. Germany was used as a high income model country. For the areas of cardiovascular and respiratory medicine density equalizing mapping procedures visualized major geographical differences in both input and output markers.

**Results:**

An imbalance in the state financial input was present with 36 cardiovascular versus 8 respiratory medicine state-financed full clinical university departments at the C4/W3 salary level. The imbalance in financial input is paralleled by an imbalance in overall quantitative output figures: The 36 cardiology chairs published 2708 articles in comparison to 453 articles published by the 8 respiratory medicine chairs in the period between 2002 and 2006. This is a ratio of 75.2 articles per cardiology chair and 56.63 articles per respiratory medicine chair. A similar trend is also present in the qualitative measures. Here, the 2708 cardiology publications were cited 48337 times (7290 times for respiratory medicine) which is an average citation of 17.85 per publication vs. 16.09 for respiratory medicine. The average number of citations per cardiology chair was 1342.69 in contrast to 911.25 citations per respiratory medicine chair. Further comparison of the contribution of the 16 different German states revealed major geographical differences concerning numbers of chairs, published items, total number of citations and average citations.

**Conclusion:**

Despite similar significances of cardiovascular and respiratory diseases for the global burden of disease, large input and output imbalances have been revealed in the present study which point to a need for changes in funding policies. The present study supplies data that could be used for decision making in the field of health systems funding.

## Background

Diseases of the cardiovascular system play an important role in health care. They have a great impact on the burden of disease. This burden of disease is defined as the impact of a health care problem in an area measured by financial cost, mortality, morbidity, or other indicators. Quantification is often performed using Disability-adjusted life years (DALYs) [1] or Quality-adjusted life years (QALYs) [2,3]. These measures combine the burden due to both death and morbidity into one index. With regard to the different disorders listed in global and national burden of disease rankings, also diseases of the respiratory play a prominent role [4]. In this respect, four out of the ten most common causes of death are respiratory diseases [5]. In view of the enormous socio-economic burden, it should be anticipated that a large proportion of health research funding is allocated to this field of medicine.

In contrast to these features, health system resources and research funding policy are often debated as being disproportional. Numerous publications discussed this issue i.e. in the field of neurosciences [6], cardiovascular medicine [7], gastroenterology [8], genetics [9] or stem cell research [10-12]. These areas are heavily funded by governmental and non-governmental sources and there are various statements concerning policy guidelines available [13-18].

For the high income country Germany, especially the fields of respiratory medicine and cardiology are interesting areas for health and research funding allocation policy. In this respect, the present study aimed to 1) identify and compare different output figures 2) relate these figures to selected input figures 3) provide data in relation to geographical information.

## Methods

### Output benchmarking data source

Data for output benchmarking (published items and citations) was retrieved from the biomedical database Web of Science (Thomson Institute for Scientific Information, ISI) [19,20].

### Search strategies

For the different searches, phrases joined together with Boolean operators, i.e. AND, OR and NOT were used.

### Time frame

A time frame was set and all entries between the years 2002 and 2006 were analysed.

### Input benchmarking data source

Data for input benchmarking was retrieved from internet searches and the German Lung White Book [21]. All full professorships/chairs (W3/C4 salary level) of medical school departments of cardiology and respiratory medicine were identified (begin of analysis 2007-08-01, last update 2008-4-30) and related to the respective German states. In this respect, the numbers of full professorships/chairs were calculated for each of the 16 German states (i.e. 3 chairs for cardiology in the state of Berlin and 0 for the state of Brandenburg) and density equalizing mapping performed. The numbers were related to each German state since German medical schools are financed directly by the single states and not by the Federal Republic of Germany. Using the calculation for each state, an input analysis was possible for cardiology and respiratory medicine.

### Output quantity analysis: Comparison of number and origin of publications in relation to the field

To perform a comparison between respiratory medicine and cardiology, published items were screened. All full professors/chairmen of medical school departments of cardiology and respiratory medicine were identified by name and their publications between 2002 and 2006 were recorded (begin of analysis 2007-08-01, last update 2008-4-30). For density equalizing procedures, only the publication type "article" was used. The entries of all full professors/chairmen for each of the 16 German states were added in order to establish a formula for each of the 16 German states for geographical distribution.

### Output quality analysis: Comparison of citations in relation to the field

To perform a qualitative comparison between respiratory medicine and cardiology, published items were related to their citations. Parallel to the quantity analysis, all full professors/chairmen of medical school departments of cardiology and respiratory medicine were identified by name and the numbers of citations of their publications between 2002 and 2006 were recorded (begin of analysis 2007-08-01, last update 2008-4-30). For density equalizing procedures, only the publication type "article" was used. The citations of all full professors/chairmen of each state were added in order to establish a formula for each of the 16 German states for geographical distribution.

### Density-equalizing mapping

The method of density-equalizing mapping was used following a recently described method [22] basing on Gastner and Newman's algorithm [23]. In brief, territories were re-sized from the original size (additional file 1) according to a particular variable, i.e. the number of published items or the citations. For the re-sizing procedure the area of each state was scaled in proportion to its total number of published items or citations.

## Results

### Input benchmarking: Spatial distribution of full professorships/chairs

**Figure 1:**
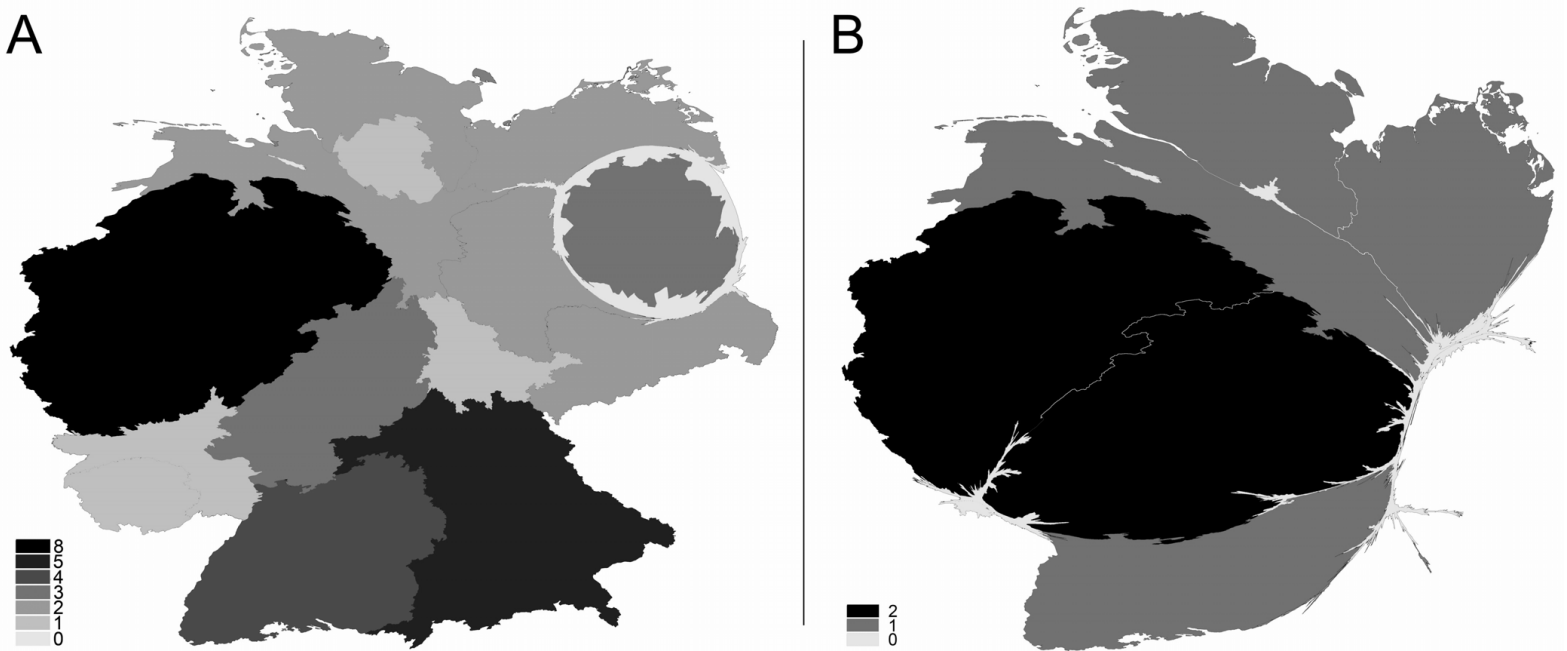
**Density equalizing mapping of full professorships/chairs in relation to single German states in 2008. **Cardiology (A) vs. Respiratory Medicine (B). Greyscales encode number of professorships per state.

Large differences were found in the input benchmark "number of full professorships/chairs" (highest level – W3/C4) of cardiology versus respiratory medicine. For cardiology, it was found that the total number of 34 medical school/faculties in Germany have established 36 full professorships/chairs (W3/C4 salary level) of cardiology. In this respect, the medical faculty of the Charité in Berlin has three independent departments of cardiology that are directed by three separate full professorships/chairs of cardiology. Also, Munich has two full professorships/chairs of cardiology which belong to two separate medical faculties. Every other university has an own department of cardiology (Fig. 1a). Density equalizing mapping calculations visualizes the geographical distribution. Every state apart from Brandenburg and Bremen has financed a medical school department for cardiology. In these states, there are no medical schools (Fig. 1a).

In contrast to these input figures, the area of respiratory medicine is represented by only 8 independent clinical full professorships/chairs (W3/C4 level) of respiratory medicine (fig. 1b). Two out of the eight are situated in Hesse at the same faculty of medicine (Justus-Liebig-University Giessen). The following states do not finance an independent full clinical professorship/chair of respiratory medicine: Berlin, Brandenburg, Bremen, Hamburg, Bavaria, Saxony, Saxony-Anhalt, Thuringia which leads to a major distortion in the density equalizing map (fig. 1b).

### Quantity output benchmarking: Total numbers and spatial distribution of published items per state

The comparison of respiratory medicine and cardiology concerning the benchmark of total numbers of published items of full professors of each German state demonstrated large quantitative differences between the two fields of medicine and the different states.

**Figure 2:**
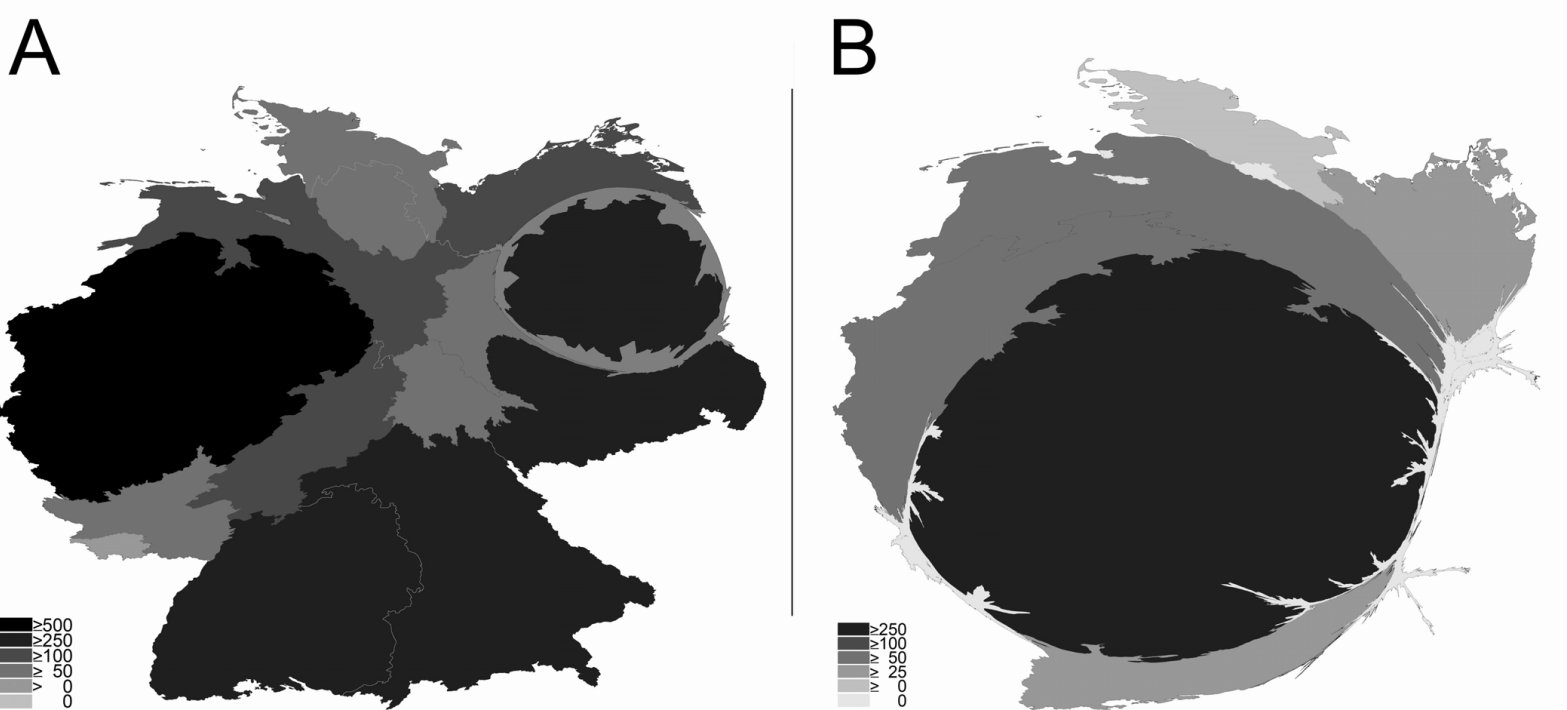
**Density equalizing mapping of total numbers of published items of the full professorships (C4/W3) per German state between 2002 and 2006.** Cardiology (A) vs. Respiratory Medicine (B). Greyscales encode total numbers of published items per state in the publication category "article".

For the German full professorship of clinical cardiology, an overall number of 2708 published items was found. The ranking was headed by North Rhine-Westfalia (#1 with 610 published items), followed by Bavaria (#2 with 413), Baden-Württemberg (#3 with 369), Berlin (#4 with 292), Saxony (#5 with 219), Lower Saxony (#6 with 163), Hesse (#7 with 134), Mecklenburg-Western Pomerania (#8 with 122), Hamburg (#9 with 88), Rhineland-Palatinate (#10 with 86), Schleswig-Holstein (#11 with 72), Thuringia (#12 with 69), Saxony-Anhalt (#13 with 60) and Saarland (#14 with 11). The states Brandenburg and Bremen do not have a medical faculty. Density equalizing mapping approaches were used to analyse the distribution and it was found that the states Lower Saxony, Saxony-Anhalt, Bremen and Brandenburg were distorted (Fig. 2a) in comparison to their natural shape (additional file 1).

For respiratory medicine, lower numbers were recorded in general with an overall number of 453 published items for all 8 chairs. In this field, Hesse led the field with a total number of 255, followed by Lower Saxony (66), North Rhine-Westfalia (52), Mecklenburg-Western Pomerania (36), Baden-Württemberg (25), and Schleswig-Holstein (19). The states Bavaria, Berlin, Hamburg, Rhineland-Palatinate, Saarland, Saxonia, Saxonia-Anhalt and Thuringia do not have an independent full professorship at the C4/W3 salary level despite having cardiology professorships. Bremen and Brandenburg do not have medical faculties and therefore neither cardiology nor respiratory full professorships. Density equalizing mapping calculations led to a strong distortion with Hesse and Lower Saxony dominating the map (Fig. 2b) in comparison to the natural shape (additional file 1).

### Quality output benchmarking: Citation numbers and spatial distribution of published items per state

Large differences were also present between the two fields with regard to output quality benchmarking. In this respect, the total number of citations of cardiology articles was 48337 versus 7290 citations of respiratory medicine articles. The average citation per item also differed with 17.85 for cardiology articles and 4.34 for respiratory articles.

To perform a detailed spatial comparison between respiratory medicine and cardiology, citations were related to the states of origin.

In the field of cardiology, the citation ranking was partly different from the publication number ranking: Parallel to the publication number ranking, North Rhine-Westfalia headed this analysis with 10825 citations, followed by Saxony (#2 with 8078 citations), Bavaria (#3 with 6003), Baden-Württemberg (#4 with 5499), Lower Saxony (#5 with 4486), Berlin (#6 with 4351), Mecklenburg-Western Pomerania (#7 with 1826), Rhineland-Palatinate (#8 with 1695), Hesse (#9 with 1653), Schleswig-Holstein (#10 with 1585), Hamburg (#11 with 1066), Saxony-Anhalt (#12 with 469), Thuringia (#13 with 464) and Saarland (#14 with 337). The states Brandenburg and Bremen do not have a medical faculty.

**Figure 3:**
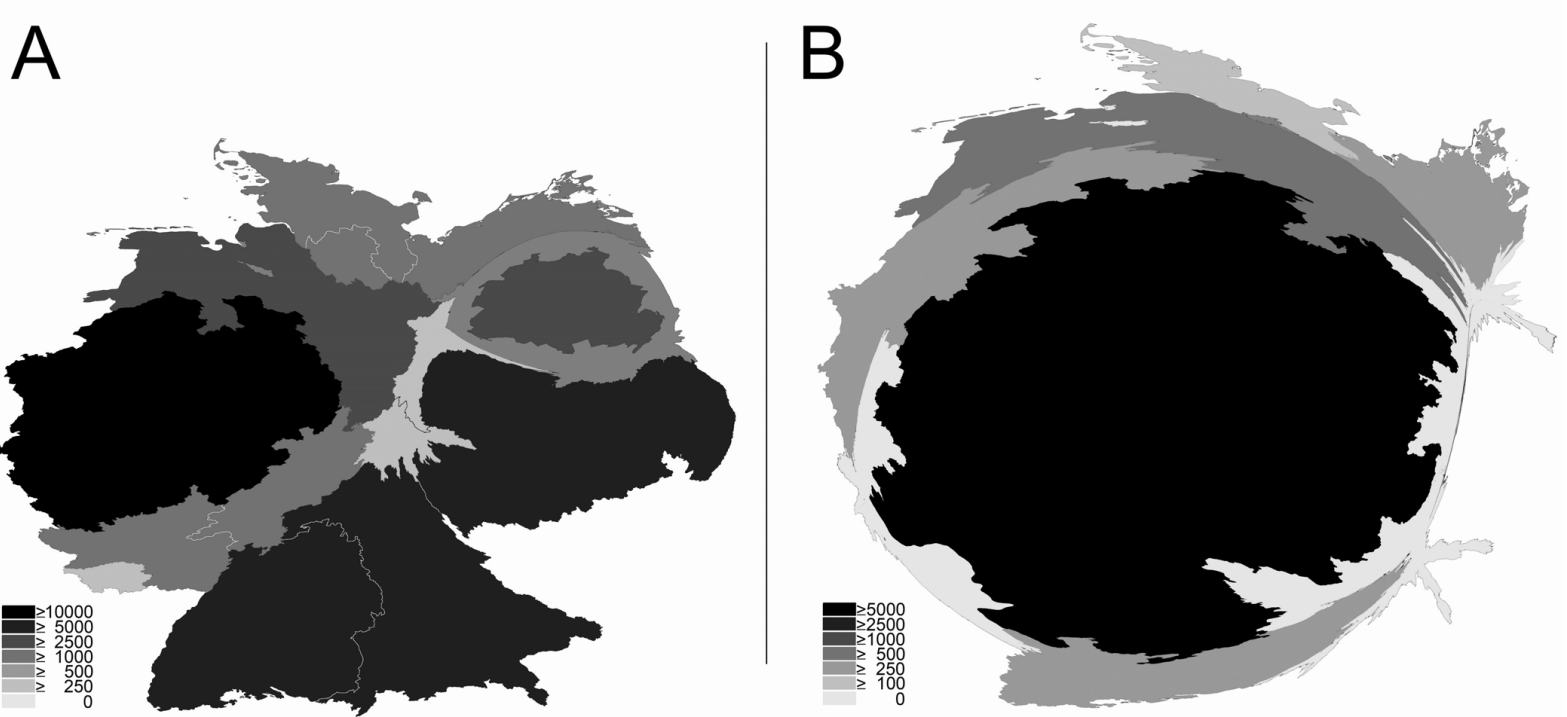
**Density equalizing mapping of citation numbers of the full professorships (C4/W3) per German state between 2002 and 2006.** Cardiology (A) vs. Respiratory Medicine (B). Greyscales encode total numbers of citations per state in the publication category "article".

Density equalizing mapping calculations to led slight distortions of the map (Fig. 3a) in comparison to the natural shape (additional file 1).

For respiratory medicine, lower numbers of citations were recorded in general with an overall number of 7290 citations for all 8 chairs.

In specific, Hesse ranked #1 with 5442 citations, followed by Lower Saxony (#2 with 682), North Rhine-Westfalia (#3 with 357 citations), Baden-Württemberg (#3 with 335), and Schleswig-Holstein (#4 with 172). The other states Bavaria, Berlin, Hamburg, Rhineland-Palatinate, Saarland, Saxonia, Saxonia-Anhalt and Thuringia do not have an independent full professorship at the C4/W3 salary level despite having cardiology professorships. Density equalizing mapping calculations again led to strong distortions in the map with Hesse dominating (Fig. 3b) in comparison to the natural shape (additional file 1) and to the shape in the cardiology citation density-equalizing map (Fig. 3a).

### Quality output benchmarking: Spatial distribution of average citations per item

Density equalizing mapping approaches were also used to assess differences in the spatial distribution of average citations per item in both fields. These calculations based on the number of publications and citations in relation to the states of origin.

**Figure 4:**
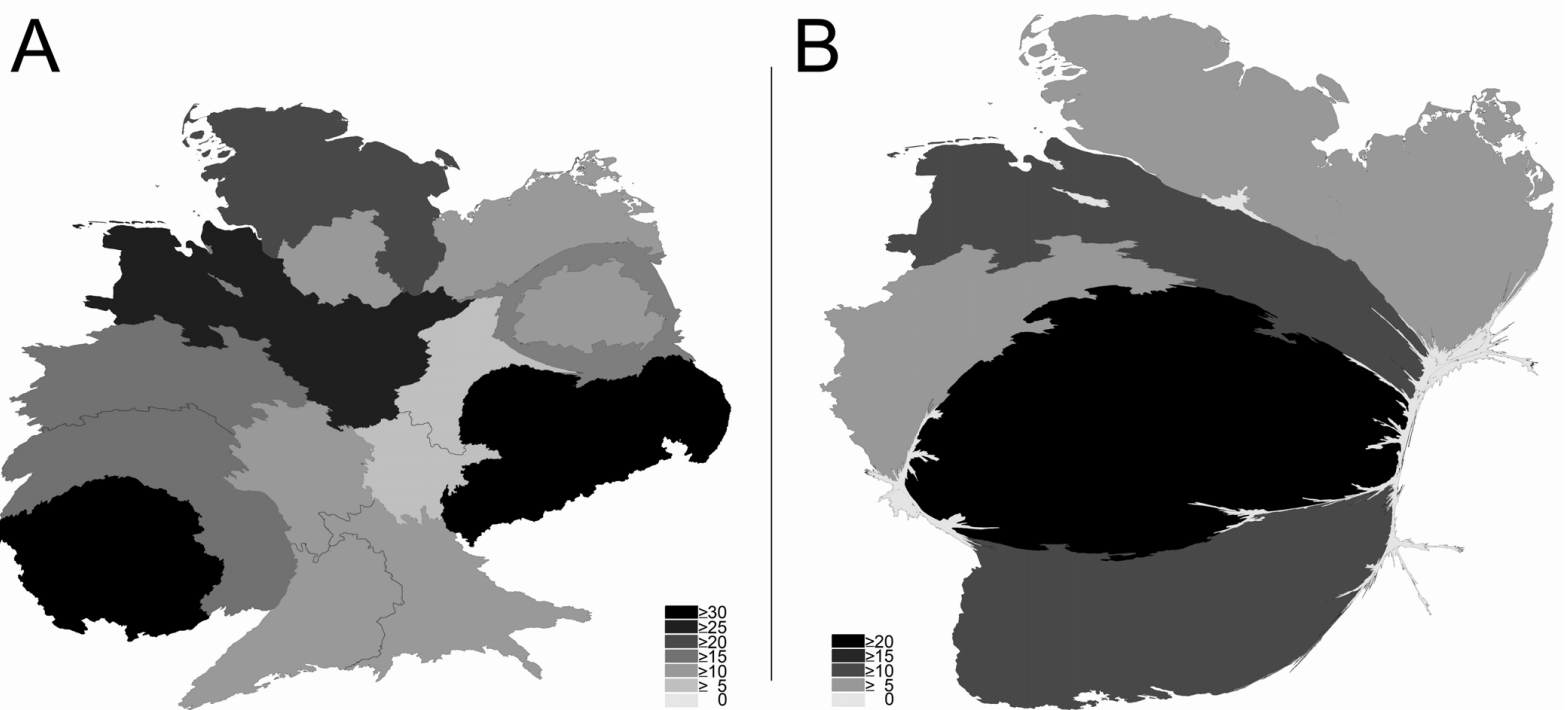
**Density equalizing mapping of average citations per published item of the full professorships (C4/W3) per German state between 2002 and 2006.** Cardiology (A) vs. Respiratory Medicine (B). Greyscales encode average citations per article per state in the publication category "article".

In the field of cardiology, the calculations led to stronger distortions in the density-equalizing map than in the publication and citation number analysis (Fig. 4a). In this respect, the average citation per item analysis listed Saxony at the first position (36.89 citations per published item) followed by Saarland (30.64), Lower Saxony (27.52), Schleswig-Holstein (22.01), Rhineland-Palatinate (19.71), North Rhine-Westfalia (17.75), Mecklenburg-Western Pomerania (14.97), Berlin (14.9), Baden-Württemberg (14.9), Bavaria (14.54), Hesse (12.34), Hamburg (12.11), Saxony-Anhalt (7.82), Thuringia (6.72). Thus, the density equalizing map was dominated by Saxony and Saarland (Fig. 4a) in comparison to the natural shape (additional file 1).

For respiratory medicine, Hesse was also ranked on first position in this analysis (Fig. 4b) with an average citation per published item of 21.34. Hesse was followed by Baden-Württemberg (#2 with 13.4 citations per item), Lower Saxony (#3 with 10.33), Schleswig-Holstein (#4 with 9.05), Mecklenburg-Western Pomerania (#5 with 8.39) and North Rhine-Westfalia (#6 with 6.87).

## Discussion

Numerous publications indicate that current settings for health system and research funding need review. Reasons are potential imbalances in the existing policy for funding allocation. The present study addressed this issue using Germany as a model high income country and the two socio-economic important fields of cardiovascular and respiratory medicine.

Methodologically, we used both output and input benchmarking. Output benchmarking was divided into the quantitative measure of total number of published items (publications type "article") and the qualitative measure of citations. The later feature is partly debated as not being a very good tool to assess research quality [24,25] but other tools such as the H-index also bear limitations [26].

In terms of input parameters, the present study is limited to the number of full professorships/chairs for cardiology vs. respiratory medicine per state. For Germany, this can be used as an indicator for governmental funding since the states are responsible for the financial support of the medical schools. In this respect, the state ministers for research and education are usually also responsible to establish full professorships/chairs. A further useful figure would have been to assess the funding for the two fields by federal funding institutions such as the German Research Council (DFG), the federal ministry for Education and research (BMBF) or the European Union and the industry [27,28]. However, the precise funding from these sources is not accessible since some institutions and departments do not uncover these figures. In specific, industry funding is often not published as demonstrated by the tobacco industry funding policies [29,30]. Therefore, the present study was limited to monitor only the state financial input in terms of established independent full professorships/chairs at medical faculties.

A further potential bias within the methodology of the present study is related to the issue of linguistic differences as previously discussed [31]. In this respect, the present analyses encompassed all languages included in the data bases. The majority of publications is published in English and it is difficult for non-English journals to get included in the data bases. Therefore, numerous scientific publications in languages other than English are not accessible. However, the major German cardiovascular (Z Kardiol – Clinical Research in Cardiology) and respiratory journals (Pneumologie) are included in the data base. Also, it is generally accepted that German scientists publish their high quality research in scientific journals that use English as language.

Large differences were present between the two fields: All medical faculties had chairs for cardiology. At the Berlin medical faculty, three cardiology chairs were present but not an independent single chair for respiratory medicine. This field was subordinated and headed by a full professorship for cardiology and a full professorship for infectious diseases. The presence of three independent cardiology chairs in Berlin is most probably due to historical reasons since this faculty was divided into two faculties during period of the Berlin wall and reunified in 2002/2003 [32].

In striking contrast to the high number of cardiology chairs, only 8 chairs for respiratory medicine were present in Germany. The regional distribution as assessed by density equalizing mapping demonstrated a focus in North Rhine-Westfalia and Hesse. The largest state Bavaria did not have a chair of respiratory medicine.

After the demonstration of an imbalance in the financial input (36 cardiology versus 8 respiratory medicine state-financed clinical university departments), the present study aimed to analyze potential imbalances in output figures. Therefore, the quantitative measure "number of publications" and the qualitative measures of "overall numbers of citations" and "average citations per published item" were used. In general, the imbalance in financial input is paralleled by an imbalance in overall quantitative output figures. I.e. the 36 cardiology full professorships published 2708 articles in comparison to 453 articles published by the 8 respiratory medicine full professorships. This is a ratio of 75.2 articles per cardiology chair and 56.62 articles per respiratory medicine chair. A similar trend is also present in the qualitative measures. Here, the 2708 cardiology professorship publications were cited 48337 times which is an average citation of 17.85 per publication. The average number of citations per cardiology chair was 1342.69. For respiratory medicine, the 453 publications were cited 7290 times. This is an average citation number of 16.09 per publication and a ratio of 911.25 citations per respiratory medicine chair.

Interestingly, the citations per state and the number of publications per state varied to a large extend between the different states and the two fields of internal medicine. For respiratory medicine, the maximal number of publications per state was 255 for the 2 chairs in Hesse. These 255 publications were cited 5442 times which is an average citation per published item of 21.34. This is a ratio of 127.5 publications per professorship in Hesse and a ratio of 2721 citations per professorship in Hesse. By contrast, the best ratios in the field of cardiology were found for Saxony. Here, the ratio of publications per professorship was 109.5 and the ratio of citations per professorship was 4039.

Closer analysis revealed that the most cited publications for the Saxony cardiology professorships were articles in which the full professor was co-author [33] whereas the most cited publications for the Hesse respiratory medicine professorships were senior authorships [34-36].

The reasons for the presently analyzed imbalances are numerous: I.e. the high income country Germany is known to have an extremely low ratio of respiratory physicians in comparison to other European countries (as indicated in the European Lung White Book [37]. Therefore, a lower number of respiratory specialists may lead to a lower research activity. 2) The number of full professorships and department chairs for respiratory medicine at the highest level (C4/W3) is disproportional in Germany in comparison to other countries since there are 36 chairs for cardiology but only 8 for respiratory medicine. This imbalance leads to a lower research activity with a lower number of publication entries in the database.

An important issue is related to the reason for this difference of 8 vs. 36 university chairs at German medical schools. Two major reasons may account for the imbalance: 1) History: in the times of tuberculosis at the beginning of the 20^th ^century, respiratory disorders were treated in remote hospitals but not in university medical schools. When the faculties started to create new chairs for internal medicine after the second world war, respiratory medicine capacities was not present at the medical faculties but in remote hospitals and the denomination of the chairs was directed towards cardiology. As a result, respiratory medicine is now underrepresented at German faculties in comparison to i.e. the UK. 2) Economics: Interventional and diagnostic procedures in cardiology such as left heart catheter offer a larger financial benefit to the faculties than respiratory interventional and diagnostic procedures [38,39]. Therefore, economic features may direct the faculties to the direction of cardiology professorships. Future studies should analyse these imbalances in closer detail.

It is difficult to interpret how input imbalance affects on the output ratios. I.e. the allocation of public and private funding to a specific field such as cardiology and the consecutive concentration of financial resources in specific fields may lead to an increase of research actors or promotion of networking between outside institutes in this area. This may then lead to increased research activities resulting in production of higher-impact publications, eventually, obtaining more funding. Eventually a circle structure may appear that leads to the phenomenon that the rich areas automatically get richer [40].

In conclusion, the present study used input and output benchmarking in combination with density equalizing mapping to assess differences in the two important fields of cardiovascular and respiratory medicine. Germany was used a model high income country. A major imbalance in the state financial input was present with 8 respiratory medicine versus 36 cardiovascular state-financed full clinical university departments at the C4/W3 salary level. This difference in the state financial input was paralleled by large differences in overall quantitative output figures with 2708 published cardiology articles in comparison to 453 respiratory medicine in the period between 2002 and 2006. However, there was also a difference between the two fields in the qualitative citation analysis. Here, cardiology publications had an average citation of 17.85 per publication whereas the respiratory medicine publication had an average citation of 16.09 per publication. This small difference might be due to the fact that a larger number of professorships lead to a larger number of networking collaborations and citations. Despite a high significance of both cardiovascular and respiratory diseases for the burden of disease, large differences are present in Germany. This should be realized for health policy and research funding allocation.

## Competing interests

The authors declare that they have no competing interests.

## Authors' contributions

BGK, AF, TW, DQ, and CS contributed to the conception and design of the study, BGK and CK performed the analysis, BGK and CS prepared the first draft and all authors contributed to the writing of the manuscript.

## Supplementary Material

Additional file 1 Map of the 16 German states. This geographic map of the 16 German states and their geographical position in Europe can be used as a matrix for the comparison with the density equalizing mappings in figures 1, 2, 3, 4. Click here for file
