# Supplementary figures and images for: Interfield dysbalances in research input and output benchmarking: Visualisation by density equalizing procedures
Source: Int J Health Geogr. 2008 Aug 25;7:48. doi: 10.1186/1476-072X-7-48 (PMC2533656; doi:10.1186/1476-072X-7-48)

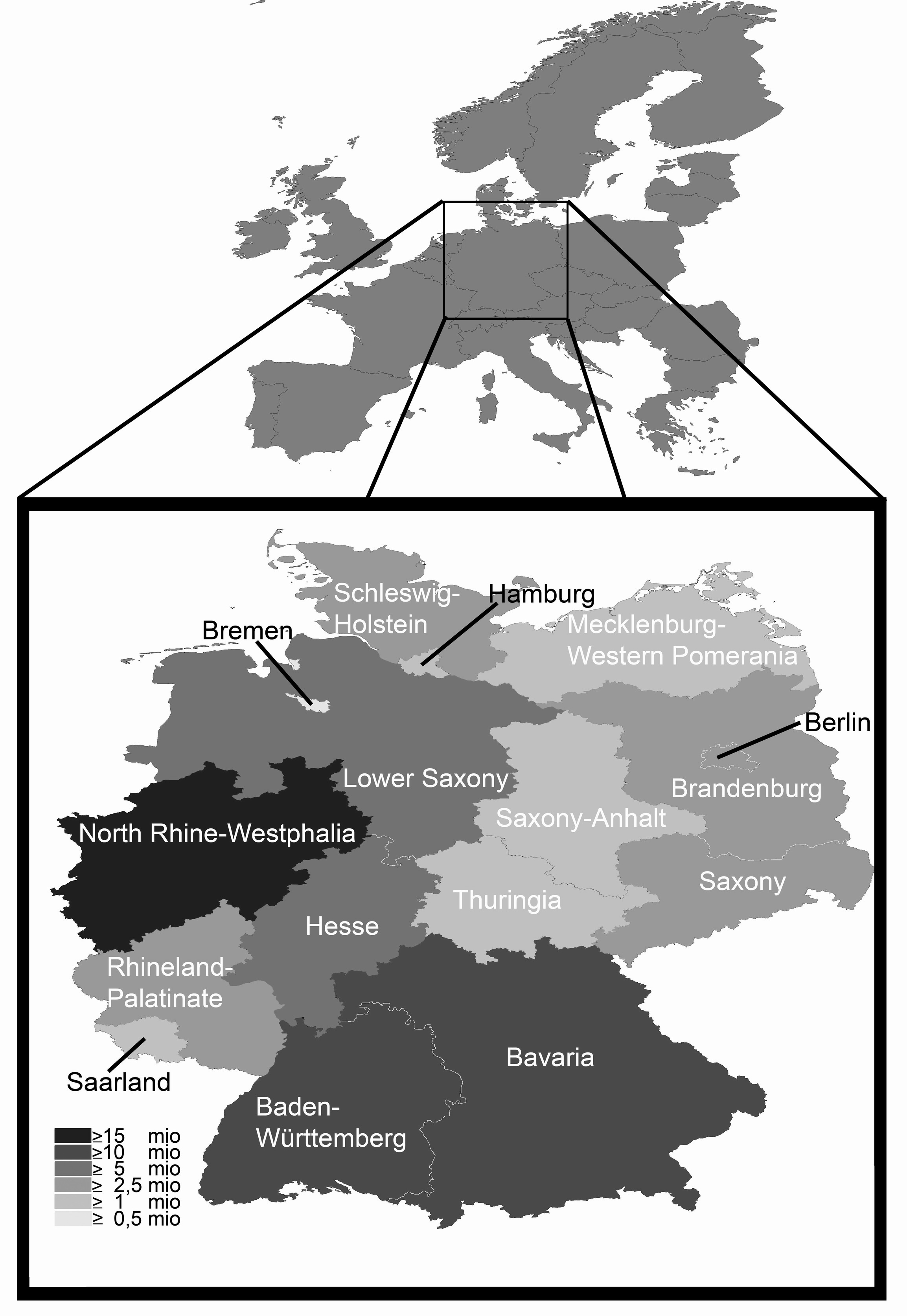

Supplement: Additional file 1 — Map of the 16 German states. This geographic map of the 16 German states and their geographical position in Europe can be used as a matrix for the comparison with the density equalizing mappings in figures 1, 2, 3, 4. [file 1476-072X-7-48-S1.jpeg]
